# In Vivo Imaging of Cone Photoreceptors in Mucopolysaccharidosis Type II and Retinal Degeneration: A Case Report

**DOI:** 10.1159/cop/acaag022

**Published:** 2026-09-11

**Authors:** Juliane Matlach, Lucas Wipprecht, Anna J. Schmelmer, Julia V. Stingl, Susanne Hopf, Susanne Pitz, Lisa Maria Maiurano, Julia B. Hennermann

**Affiliations:** a Department of Ophthalmology, University of Leipzig, Leipzig, Germany; b Department of Ophthalmology, University Medical Center of the Johannes Gutenberg University of Mainz, Mainz, Germany; c Department of Ophthalmology, Orbitazentrum, Bürgerhospital Frankfurt, Frankfurt, Germany; d Center for Pediatric and Adolescent Medicine, Villa Metabolica, University Medical Center of the Johannes Gutenberg University of Mainz, Mainz, Germany

**Keywords:** High magnification module, Cone photoreceptors, Mucopolysaccharidosis, Optical coherence tomography angiography, Case report

## Abstract

**Introduction:**

Mucopolysaccharidosis type II (MPS II, Hunter syndrome) is a rare X-linked lysosomal storage disorder frequently associated with progressive pigmentary retinopathy and retinal degeneration. High-resolution, noninvasive imaging techniques have expanded our ability to assess photoreceptor integrity and retinal microvasculature in vivo. This study evaluates multimodal retinal imaging, including the High Magnification Module (HMM^®^) without adaptive optics (AO), in adults with MPS II.

**Case Presentation:**

Three adult patients aged 22–45 years with genetically confirmed MPS II and pigmentary retinopathy underwent multimodal retinal imaging using spectral-domain optical coherence tomography (SD-OCT; Spectralis^®^ OCT), OCT angiography (OCT-A), and the HMM^®^ add-on lens. SD-OCT demonstrated perifoveal outer retinal atrophy, characterized by attenuation of the ellipsoid zone and thinning of the outer nuclear layer. In 1 patient with successful OCT-A image acquisition, OCT-A revealed reduced capillary density within regions corresponding to outer retinal atrophy, indicating associated microvascular compromise. In 1 patient with successful HMM image acquisition, high-magnification imaging with the HMM, performed without AO, showed clear rarefaction of cone photoreceptors in areas of structural loss, providing direct cellular-level insight into photoreceptor degeneration. The imaging quality in this patient allowed visualization of the cone mosaic despite the absence of AO.

**Conclusion:**

Multimodal retinal imaging combining SD-OCT, OCT-A, and HMM without AO may provide complementary information on photoreceptor structure and retinal microvasculature in patients with MPS II. This case series provides a proof-of-concept for the feasibility of HMM imaging in 1 patient for in vivo visualization of the cone mosaic in MPS II and highlights limitations in image acquisition in patients with more advanced retinal degeneration.

## Introduction

Mucopolysaccharidoses (MPSs) are a group of rare, inherited lysosomal storage disorders resulting from deficient activity of specific lysosomal enzymes, leading to glycosaminoglycan (GAG) accumulation in various tissues. The severity of ocular involvement varies across MPS types but commonly includes corneal clouding, glaucoma, strabismus, refractive errors, and pigmentary retinopathy [1–6]. Pigmentary retinopathy, characterized by retinal pigment epithelium (RPE) and photoreceptor loss, has been reported in MPS types I, II, III, and IVA [1, 4]. Histopathological studies in MPS II confirm outer retinal and RPE degeneration [7]. Advances in ocular imaging have enabled noninvasive, high-resolution visualization of retinal microstructures. Spectral-domain optical coherence tomography (SD-OCT) has revolutionized the diagnosis and monitoring of retinal diseases, including inherited metabolic disorders such as MPS, by revealing characteristic outer retinal thinning and ellipsoid zone disruption in certain MPS types [3, 8]. Using SD-OCT, Seok et al. [8] demonstrated photoreceptor degeneration characterized by thinning of the parafoveal and perifoveal ellipsoid zones, thickening of the external limiting membrane (ELM), and RPE alterations in patients with MPS I and II, consistent with progressive retinal degeneration [8].

Additional imaging techniques, such as OCT angiography (OCT-A), provide a dye-free assessment of retinal capillary density, while adaptive optics (AO) enables visualization of individual photoreceptors but remains technically complex and less widely available [9, 10]. The High Magnification Module (HMM^®^; Heidelberg Engineering) lens is a practical alternative to AO, offering ∼1.5-μm-pixel resolution across an 8° field. Integrated with the Spectralis^®^ OCT platform, HMM allows in vivo imaging of the cone photoreceptor mosaic without the use of AO and can be used to detect cone loss in retinal diseases [11].

This report presents multimodal imaging findings in 3 MPS II patients, illustrating the clinical potential of HMM and OCT-A in evaluating retinal degeneration in lysosomal storage diseases. To our knowledge, this is the first report of HMM use in patients with MPS-related retinal degeneration.

## Case Presentation

Three male patients with MPS type II, aged 22, 25, and 46 years, presented with varying degrees of pigmentary retinopathy. Intraocular pressure was within normal limits, and anterior segment examination was unremarkable except for mild stromal corneal clouding in the 25- and 46-year-old patients. Fundus examination revealed widespread pigmentary changes consistent with pigmentary retinopathy. The 46-year-old patient exhibited more advanced pigmentary retinopathy compared to the younger individuals, suggesting a progressive disease course. Static automated perimetry was performed with an Octopus 900 perimeter (Haag-Streit AG, Köniz, Switzerland) using the 30° G-Dynamic program (standard white-on-white perimetry, Goldmann size III stimulus), demonstrating early concentric visual field loss in the 22-year-old patient, with a developing temporal scotoma extending to approximately 10°. In contrast, the 25- and 46-year-old patients exhibited more advanced concentric visual field constriction, with residual visual fields of approximately 5–10° (Fig. 1). Clinical characteristics, including ophthalmic findings, are summarized in Table 1.

**Fig. 1.**
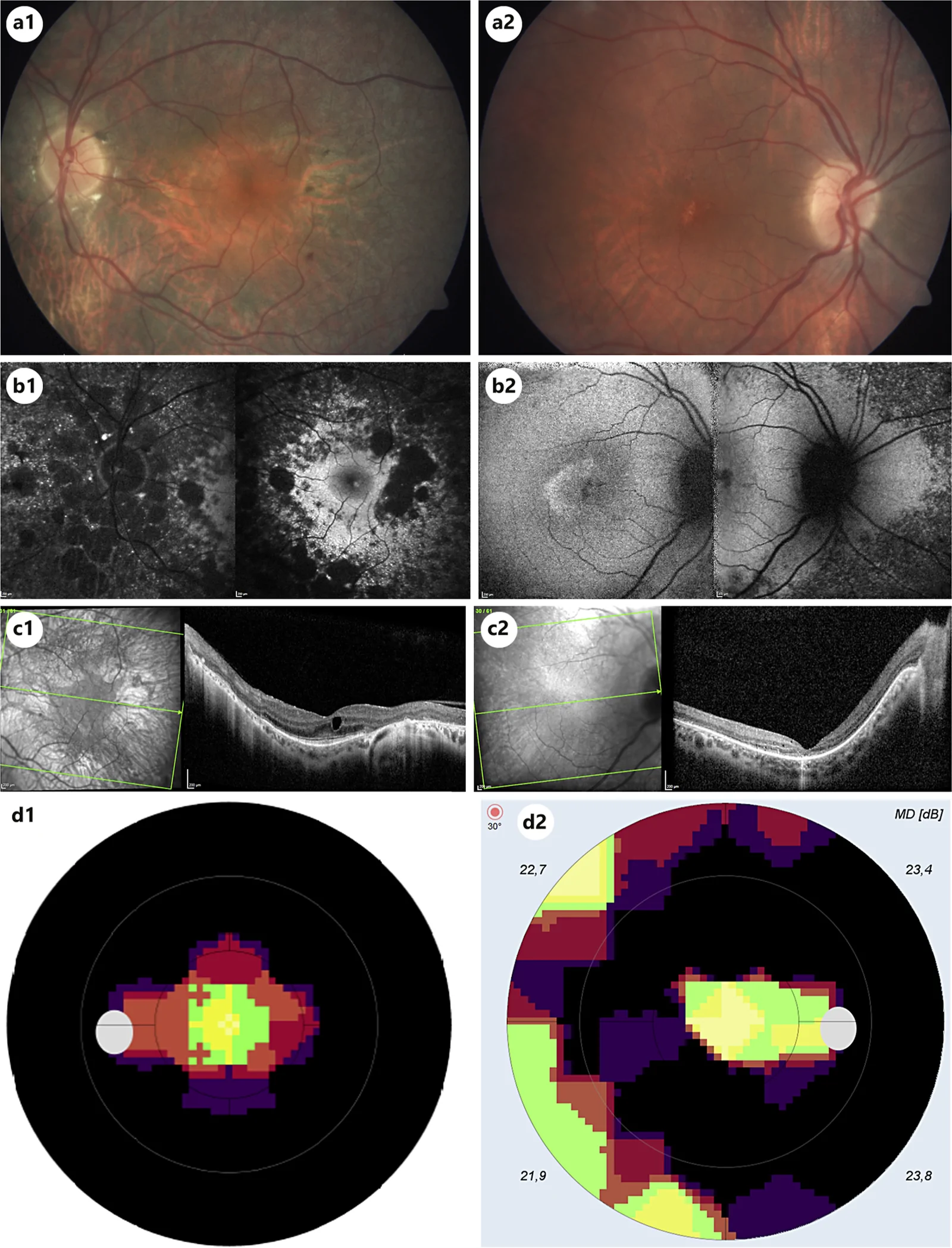
Multimodal retinal imaging in MPS II. Fundus photographs show a salt-and-pepper appearance of the retinal pigment epithelium (RPE) and mildly pale optic disks in the 46-year-old patient (**a1**), with less pronounced changes in the 25-year-old patient (**a2**). Fundus autofluorescence reveals widespread hypoautofluorescence at the posterior pole, with patchy signal loss around the macula and optic disk (**b1**, **b2**). Spectral-domain optical coherence tomography (SD-OCT) shows diffuse outer retinal atrophy, including photoreceptor and RPE loss, a relatively preserved fovea with a thickened ELM, and intraretinal cysts (**c1**, **c2**). These findings indicate advanced pigmentary retinopathy in the 46-year-old patient (**a1–c1**), with milder changes in the 25-year-old MPS II patient (**a2–a2**). Visual field testing reveals concentric field loss on automated static 30° perimetry (**d1**, **d2**). For illustrative purposes, 1 eye from each patient was selected to demonstrate retinal changes. Both eyes of the 2 patients with MPS II have moderate-to-severe pigmentary retinopathy.

**Table 1.** Overview of clinical findings

​	Patient 1	Patient 2	Patient 3
Age at examination, years	22	25	46
MPS II form	Non-neuronopathic	Non-neuronopathic	Non-neuronopathic
Residual iduronate-2-sulphatase activity	2%	0%	n.a.
Mutation in *IDS* gene	c.240+2T>A	c.1389G>A	n.a.
Spherical error (OD/OS)	+2.00/+2.25	+6.75/+8.00	+5.50/+5.25
Astigmatism (OD/OS)	−0.75/-1.50	−0.50/-0.50	−0.50/-0.25
VA decimal (OD/OS)	0.5/0.63	0.5/0.7	0.4/0.8
Corneal clouding	−	+	+
Optic nerve atrophy	+	+ to ++	+++
Pigmentary retinopathy	+	+ to ++	+++
VF concentric defects	+	++	+++
FAF macular hypofluorescence	−	+ to ++	+++
OCT macular outer retinal atrophy	+	++	+++
OCT reduced pRNFL	+	++	+++
OCT-A reduced capillary density	+	*Not possible*	*Not possible*
HMM irregular cone photoreceptor mosaic	+	*Not possible*	*Not possible*

− none; + mild/early; ++ moderate; +++ severe. FAF, fundus autofluorescence; OCT, optical coherence tomography; HMM, high magnification module; pRNFL, peripapillary retinal nerve fiber layer; OCT-A, OCT, angiography; OD, oculus dexter; OS, oculus sinister; n.a., not available.

Multimodal imaging included color fundus photography, fundus autofluorescence, OCT, OCT-A, and imaging with the HMM. Fundus autofluorescence revealed hypoautofluorescence at the posterior pole with patchy lesions around the macula corresponding to areas of RPE atrophy. OCT showed diffuse atrophy of the photoreceptor and RPE layers, a relatively preserved fovea, a thickened ELM, and multiple cystoid spaces in the inner retina (Fig. 1). HMM imaging demonstrated areas of cone photoreceptor loss, with a sparse or disrupted cone mosaic and a diminished or irregular photoreceptor pattern. OCT-A revealed reduced capillary density in the superficial retinal vascular plexus, corresponding to areas of outer retinal degeneration (Fig. 2). Not all examinations, particularly OCT-A and HMM imaging, could be performed in all patients because image quality was insufficient in the 2 patients with advanced retinal degeneration. The 2 older patients (25 and 46 years old) exhibited mild stromal corneal clouding, which did not substantially affect image acquisition. Therefore, the reduced image quality was primarily attributable to retinal degeneration rather than corneal opacity.

**Fig. 2.**
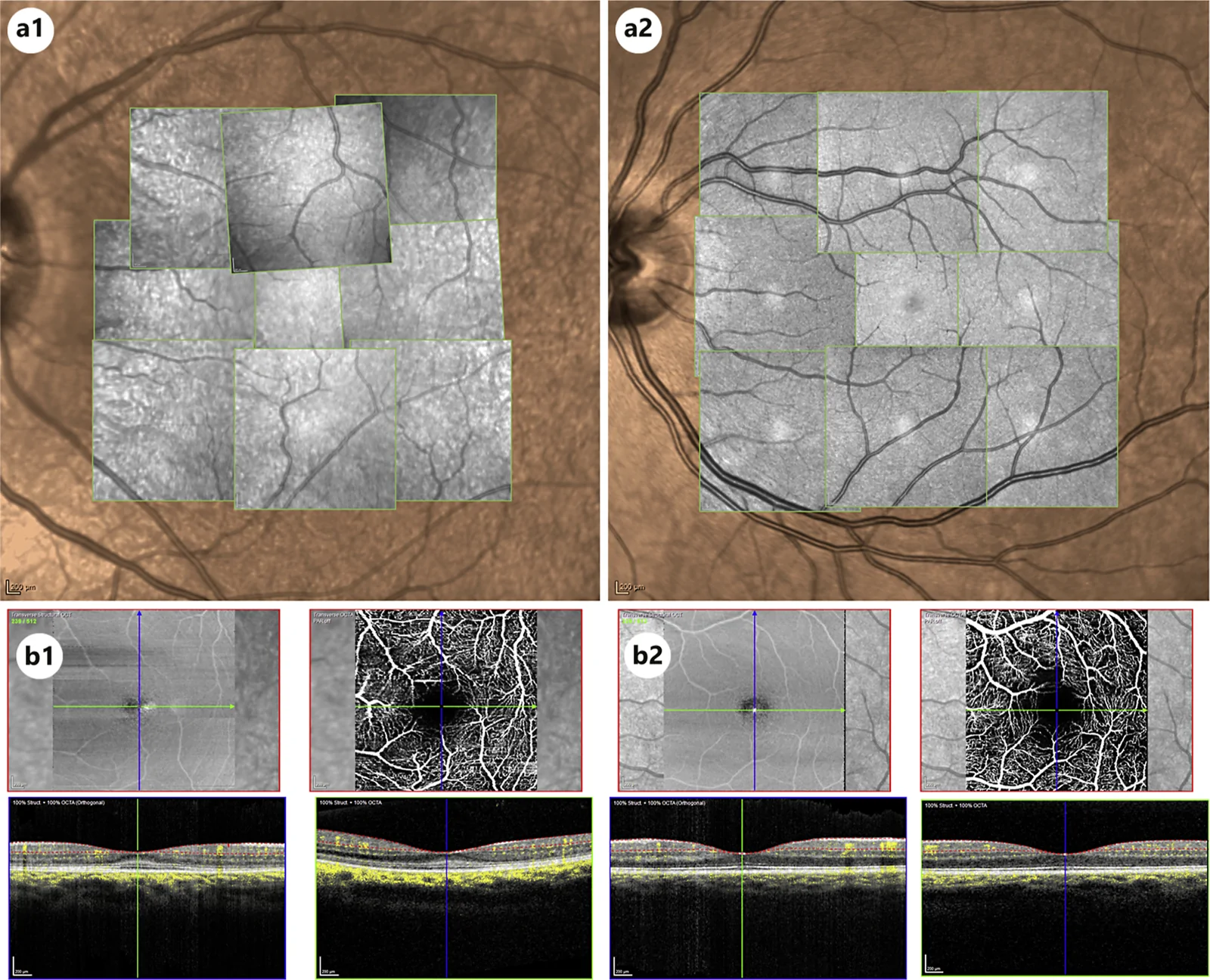
High Magnification Module (HMM) image and OCT angiography (OCT-A) in MPS II. HMM reveals an irregular cone photoreceptor mosaic in atrophic areas in a 22-year-old MPS II patient (**A1**), corresponding to reduced capillary density on OCT-A (**B1**), compared to an age-matched healthy control (**A2**, **B2**).

## Discussion

Ocular manifestations in MPS vary by subtype but commonly include corneal clouding, optic nerve abnormalities, and pigmentary retinopathy resulting from RPE and photoreceptor degeneration. In MPS II, histopathological studies have demonstrated significant degeneration of the RPE and outer retina. Retinal changes in MPS resemble those seen in retinitis pigmentosa, characterized by photoreceptor and RPE loss. In MPS, primary lysosomal storage of GAGs and their degradation products is accompanied by secondary and tertiary cellular alterations, including impaired autophagy, disrupted mitochondrial dynamics, and inflammatory processes. The clinical cases presented here are consistent with previous reports of outer retinal thinning and pigmentary alterations in MPS II. Choroidal thinning, particularly in depigmented regions, may be secondary to GAG accumulation in the sclera and adjacent tissues, contributing to outer retinal atrophy. The ELM may appear thickened and irregular, possibly reflecting Müller cell activation due to retinal stress or GAG deposition. Cystoid spaces in the inner retina and disruption of the ellipsoid zone may indicate a combination of reversible edema and irreversible photoreceptor damage [1, 3].

In our cohort, SD-OCT revealed outer retinal atrophy consistent with progressive photoreceptor degeneration in MPS II. Clinical imaging studies support these findings, demonstrating outer retinal thinning, disruption of the ellipsoid zone, and choroidal alterations [3, 8]. Sornalingam et al. [5] described a spectrum of retinal phenotypes in MPS ranging from mild RPE mottling to severe pigmentary retinopathy. OCT-A revealed reduced capillary density in the superficial retinal vascular plexus, corresponding to areas of retinal degeneration in 1 patient. As electroretinography was not performed, it was not possible to determine whether rod or cone dysfunction predominated. Nevertheless, the clinical findings, including concentric visual field loss and pigmentary retinopathy with outer retinal atrophy suggest predominant involvement of the peripheral retina first.

Advanced imaging techniques, such as AO, can detect early photoreceptor changes and monitor disease progression in inherited retinal disorders [9, 10]. Compared with AO, HMM may be more readily available and easier to implement in clinical practice, although it generally provides less detailed visualization of photoreceptor structures [11]. Structural abnormalities detected with conventional multimodal imaging were associated with reduced visual function and, in the patient in whom high-resolution imaging was successfully obtained, were further characterized by OCT-A and HMM imaging. Successfully acquired in 1 patient, OCT-A demonstrated reduced capillary density in the superficial retinal vascular plexus, and HMM imaging revealed a sparse and disrupted cone photoreceptor mosaic consistent with advanced outer retinal degeneration. The mechanism underlying the reduced superficial vascular density remains uncertain but may represent secondary retinal microvascular remodeling in response to advanced retinal degeneration.

In the present case series, HMM imaging was successfully acquired in only 1 of the 3 patients, reflecting the technical challenges associated with imaging eyes with advanced retinal degeneration. In this patient, HMM demonstrated a sparse and disrupted cone photoreceptor mosaic with an irregular arrangement of photoreceptors, consistent with significant outer retinal degeneration. The imaged region corresponded to an area with preserved retinal structure adjacent to zones of outer retinal atrophy identified on OCT. These findings provide a proof-of-concept for the feasibility of HMM imaging without AO for in vivo visualization of the cone mosaic in one MPS II-patient. However, due to the limited number of successfully imaged patients, we were unable to assess reproducibility or determine cone photoreceptor status across different regions and stages of chorioretinal atrophy.

## Conclusion

We report retinal findings in 3 adult patients with MPS type II, highlighting pigmentary retinopathy and outer retinal atrophy. Multimodal retinal imaging, including OCT-A and HMM, provided complementary information on retinal structural and vascular alterations in the 1 patient in whom image acquisition was successful. HMM imaging was feasible in 1 patient and revealed marked disruption of the cone photoreceptor mosaic, suggesting photoreceptor involvement beyond that detectable on conventional imaging. These findings should be considered a proof-of-concept rather than evidence of established clinical utility. Further studies are needed to determine the feasibility and reproducibility of HMM imaging across different stages of retinal degeneration and to establish its potential role in the assessment and longitudinal monitoring of retinal degeneration in MPS and related lysosomal storage disorders.

## Statement of Ethics

The study was approved by the Medical Ethics Committee of the State Chamber of Medicine of Rhineland-Palatinate in Mainz, Germany (Reference No. 2021-15589) and adhered to the tenets of the Declaration of Helsinki. Written informed consent was obtained from the patients for publication of this case report and any accompanying images. The CARE Checklist has been completed by the authors for this case report, attached as online supplementary material (for all online suppl. material, see https://doi.org/10.1159/cop/acaag022).

## Conflict of Interest Statement

Lucas Wipprecht, Anna J. Schmelmer, Julia V. Stingl, Susanne Hopf, and Lisa Maiurano declare no conflict of interest. Juliane Matlach has received honoraria for participating in advisory boards from Amgen and Chiesi. Susanne Pitz received travel and lecture honoraria from Sanofi, Argenx, and Amgen. Julia B. Hennermann has received travel expenses and/or honoraria from Amicus, Chiesi, Sanofi, and Takeda. Heidelberg Engineering has provided the HMM^®^ lens for photoreceptor imaging.

## Funding Sources

The authors declare no funding was obtained or used for this study.

## Author Contributions

J.M. performed most of the ophthalmic exams and drafted the case report, and analyzed and interpreted the collected data. S.H. and J.M. designed and wrote the ethnic proposal, reviewed by J.B.H. L.W., A.J.S., and J.V.S. performed all imaging examinations of the patients and reviewed the manuscript. S.P. critically reviewed the manuscript. L.W., A.J.S., L.M., and J.B.H. recruited the subjects. J.B.H. and L.M. collected general clinical data of the MPS patients. All made substantial contributions to the conception of the work and the analysis and interpretation of data. All authors contributed to the drafting and revision of the case report and have read, approved the final version for submission, and agreed to be accountable for all aspects of the work.

## Data Availability

All data generated or analyzed in this case report are contained within this published article. Additional information is available from the corresponding author upon reasonable request.
